# Prostate embolization: patient selection, clinical management and results

**DOI:** 10.1186/s42155-019-0049-1

**Published:** 2019-01-18

**Authors:** Shamar Young, Jafar Golzarian

**Affiliations:** Department of Radiology, Division of Interventional Radiology, University of Minnesota, 420 Delaware ST SE, Minneapolis, MN 55455 USA

**Keywords:** Prostate artery embolization, Benign prostatic hyperplasia, Lower urinary tract symptoms, International prostate symptom score

## Abstract

**Background:**

Prostate artery embolization is an emerging technique, that with the continued publication of promising data, is slowly moving from the research to the everyday clinical setting.

**Main body:**

This paper reviews the patient selection, clinical management and expected results of prostate artery embolization. Patient selection is paramount in delivering the desired results for any procedure. Likewise, the ability to clinically manage patients in the pre-operative and post-operative setting is an important skill to acquire when implementing new techniques. This paper introduces important urologic measurements/tests, patient selection paradigms, and clinical management concepts for interventional radiologists. It also reviews the outcomes patients can expect following prostate artery embolization as well as the complication profile.

**Conclusion:**

Prostate artery embolization is a promising technique for the treatment of benign prostatic hyperplasia induced lower urinary tract symptoms.

## Introduction

Lower urinary tract symptoms (LUTS) are a common issue for men, especially as they age. While there are a number of underlying causes of LUTS, the most common cause in middle age and older men is benign prostatic hyperplasia (BPH). Symptomatic BPH has been reported in as many as 430 per 1000 men aged 60–69 (Garraway et al. 1991); the overall prevalence in men aged 40 to 80 has been estimated at 33% in the USA (Girman et al. 1994). BPH is also associated with morbidity having been shown to lead to a decline in urinary associated and overall quality of life (Yoshimura et al. 2002; Hunter et al. 1995). Moreover, this condition results in significant economic burden with 8 million visits made to physicians with a primary or secondary diagnostic code of BPH in the USA during 2000 (Wei et al. 2008). The estimated cost was 1.1 billion dollars without factoring in the cost of pharmaceuticals, with up to 38 million hours in lost productivity (Wei et al. 2008).

Initial therapy for symptomatic BPH is medical, typically 5-alpha-reductase inhibitors or alpha blockers. However, for many patients, this is not sufficient and intervention is needed. Recently, prostate artery embolization (PAE) has been developed as a minimally invasive means to treat BPH related LUTS. Initial early and midterm results have been very promising (Bilhim et al. 2013a; Li et al. 2015; Pisco et al. 2013a; Pisco et al. 2011; Pisco et al. 2012; Pisco et al. 2013b; Pisco et al. 2016a; Pisco et al. 2016b; Antunes et al. 2013; Bagla et al. 2014; Ray et al. 2018; Abt et al. 2018; Grosso et al. 2015; Kurbatov et al. 2014; Wang et al. 2015; Wang et al. 2016; Amouyal et al. 2016a; Carnevale et al. 2010; Carnevale et al. 2011; Carnevale et al. 2012; De Assis et al. 2015; Gao et al. 2014; Bilhim et al. 2015; Carnevale et al. n.d.; Goncalves et al. 2016; Bilhim et al. 2013b; Yu et al. 2017; Carnevale et al. 2017; Amouyal et al. 2016b; Bagla et al. 2013; Carnevale et al. 2014). As further data is being published this exciting technique is slowly moving from the research setting to everyday clinical practice as demonstrated by its mention in the updated National Institute for Health and Care Excellence (NICE) guidelines for LUTS. Here in, the authors review the patient selection process, clinical management of, and expected results for PAE.

## Patient selection

Prostate artery embolization, similar to all other interventions for medically refractory BPH induced LUTS, is not a suitable treatment for all patients with this disease. It is important that treating interventional radiologists become familiar with several key tools and measurements. In this section we review the patient work up and selection process that can help providers achieve excellent results for there patients.

The gold standard for evaluating severity of BPH induced LUTS, as well as tracking improvement after intervention, is the International Prostate Symptom Score (IPSS). This 8 item questionnaire (Table 1) has a minimum score of 0 and maximum of 35, based on the patients’ response to the first 7 questions. While opinions vary, most providers would agree patients with a score less than 8 likely do not warrant treatment until there symptoms progress. It is important to note that the frequently discussed quality of life (QoL) measurement is based on the last question of the IPSS which ranges from a score of 0 (essentially no significant symptoms) to 6 (terrible symptoms) (Table 2). While this question does not contribute to the overall IPSS score it is considered an important measurement for both selection and follow up.

**Table 1:** International Prostate Symptom Score

In the past month	Not at all	Less than 1 in 5 times	Less than half the time	About half the time	More than half the time	Almost always
1. Incomplete Emptying^a^: How often have you had the sensation of not emptying your bladder?	0	1	2	3	4	5
2. Frequency^b^: How often have you had to urinate less than every two hours?	0	1	2	3	4	5
3. Intermittency^a^: How often have you found you stopped and started again several times when you urinated?	0	1	2	3	4	5
4. Urgency^b^: How often have you found it difficult to postpone urination?	0	1	2	3	4	5
5. Weak Stream^a^: How often have you had a weak urinary stream?	0	1	2	3	4	5
6. Straining^a^: How often have you had to strain to start urination?	0	1	2	3	4	5
	None	1 time	2 times	3 times	4 times	5 times
7. Nocturia^b^: How many times did you typically get up at night to urinate?	0	1	2	3	4	5

^a^Indicates a question which is utilized in the IPSS voiding subscore

^b^Indicates a question which is utilized in the IPSS storage subcore

**Table 2:** Quality of life aspect of the International Prostate Symptom Score

Quality of Life Due to Urinary Symptoms	Delighted	Pleased	Mostly satisfied	Mixed	Mostly Dissatisfied	Unhappy	Terrible
If you were to spend the rest of your life with your urinary condition just the way it is now, how would you feel about that?	0	1	2	3	4	5	6

All patients with an enlarged prostate and an elevated IPSS do not have LUTS because of their enlarged prostate. Specifically, patients may have co-morbid or dominant bladder disease that is responsible for their complaints. Therefore, many providers will subdivide the IPSS into the IPSS voiding subscore (IPSS-V), made up of questions 1, 3, 5, and 6 of the IPSS questionnaire and the IPSS storage subscore (IPSS-S) made up of questions 2, 4, and 7 (Table 1). At times a ratio of the IPSS V divided by the IPSS-S (IPSS V/S) will be calculated. In general, the larger the IPSS V/S ratio, the more likely it is that patients are experiencing issues as a result of obstruction (which is consistent with BPH induced LUTS). PAE should be carried out cautiously (or not at all) in patients with IPSS scores driven by the components of the IPSS-S, or those that have a low IPSS V/S ratio, as it is likely these patients suffer more from bladder pathology than outlet obstruction from an enlarged prostate. Referral to a Urologist for further work up is warranted in these cases.

Other important measurements to be familiar with and evaluate when working up a patient for PAE are patients maximal flow rate (Qmax), prostate volume (PV), prostate specific antigen (PSA), and post void residual (PVR). In general patients with small prostate glands (< 40 g) are likely not ideal candidates for PAE, although data in this area is variable. Unlike many surgical interventions large prostates appear to respond well to PAE (Kurbatov et al. 2014; Wang et al. 2015). Many authors also believe patients with Qmax > 15 mL/s should be worked up for other causes of LUTS and are unlikely to benefit from PAE. Finally, patients should be screened for prostate cancer. There are a number of algorithms which typically include risk stratification using PSA, biopsy results, etc. It is likely less important which algorithm the intervetionalist uses than it is to have an algorithm and follow it.

PAE requires arterial patency, and for this reason many centers are obtaining computed tomography angiogram (CTA) or magnetic resonance angiogram (MRA) imaging prior to scheduling the patient. These imaging studies can help identify patients with arterial occlusions that may prevent successful PAE and thus make these patients poor PAE candidates. They also allow the interventionalist to plan the procedure by identifying the origins and at times the number of prostatic arteries.

Prostate lobe volume composition and histology may play a role in success as well, although data in this area is very limited. In a retrospective review of 68 patients Little et al. demonstrated that adenomatous dominant BPH was a positive predictor of IPSS improvement (Little et al. 2017). While further data is needed before full scale practice change is made, this paper was an important step forward in thinking about patient selection. Some authors have also expressed their belief that patients with large middle lobes are not ideal candidates as compared to those with enlargement primarily occurring in the lateral lobes (Little et al. 2017). The concern is that patients with enlargement primarily occurring in the middle lobe is that a ball valve component of obstruction can occur. While further data is needed to support these beliefs it is likely worth noting the composition of hypertrophy prior to treatment.

## Clinical management

A detailed explanation of the procedure along with procedural “tips and tricks” can be found in multiple publications, and is beyond the scope of this paper (Sabharwal and Popert n.d.; Carnevale and Antunes 2013). Instead we will focus on the pre and post procedural clinical management in this section.

At our institution an interventional radiologist will see all patients in clinic prior to PAE. We hope to have basic testing, such as urodynamics, PSA, CTA/MRA, and IPSS completed prior to the visit, however, this is not always possible. We always perform an IPSS at the initial consultation and review BPH induced LUTS specific medications and medication history. This is to ensure the patients have undergone and failed appropriate medical management, an important prerequisite, prior to offering them PAE. If the patient is deemed a good PAE candidate and desires to proceed they are scheduled for the procedure, typically within a couple weeks. Patients are given nonsteroidal anti-inflammatory drugs (NSAID) prior to their procedure and continued on them for 5–7 days after the procedure. Similarly, patients are given 400 mg of ciprofloxacin intravenously (IV) just prior to the procedure, and then 500 mg twice a day for 5–7 days after the procedure.

PAE is typically performed as an outpatient procedure, however, it is important to warn patients about possible complications, and also for the intervetnionalist to know how to deal with these complications. The most dreaded complications are those of non-target embolization. Non-target embolization affecting the rectum, bladder, and penis have been reported (Pisco et al. 2016b; Sabharwal and Popert n.d.; Carnevale and Antunes 2013; Moreira et al. 2017; Moreira et al. 2013). While the majority of non-target embolization injuries have resolved with symptomatic treatment, a few have required surgical repair (Pisco et al. 2016b). If patients present with symptoms of rectal or bladder non-target embolization, such as hematochezia, hematuria, bladder spasms, pain on defecation, etc., they are referred for proctoscopy or cystoscopy for evaluation. Penile non-target embolization is generally seen on the skin and can be followed in that way. It is important to discuss this potential with the patient and ask them to alert you if any symptoms should arise both at the pre-PAE consultation and on the day of embolization.

Another potential complication is acute urinary retention, which has been reported as often as 8% of the time (Pisco et al. 2016b; Antunes et al. 2013; Grosso et al. 2015). Patients typically present with inability to urinate, believed to be caused by swelling of the ischemic prostate. This swelling increases the pressure in the intra-prostatic urethra and results in the patients being unable to void. Patients are treated with Foley catheter placement which can typically be removed in 1–2 weeks. Urinary tract infections are also a known complication of PAE. It is possible that these result from decreased voiding secondary to the transiently increased pressure in the intra-prostatic urethra as well. However, patients should be monitored for symptoms, especially in the period after stopping post procedural antibiotics.

Multiple other minor and self-limited complications of PAE have been reported including hematuria, hematospermia, and pain. These symptoms are typically symptomatically managed and resolve after a week or two. It is important to discuss them with the patient so they are not alarmed if they occur. We encourage patients to abstain from sexual activity for a week or two following PAE. This abstinence is primarily to allow the prostate to rest and avoid trauma to this region.

Follow up schedules vary among practices. Our practice follows patients up at 1, 3, 6, and 12 months when not on a study protocol. At these visits patients always undergo IPSS evaluations. Non-research patients do not always undergo repeat urodynamic testing, unless clinically indicated. Similarly, follow up imaging is often not needed in patients not on research protocol, unless clinical concerns dictate further evaluation. Because the prostate gland has to undergo infarction and remodeling in order to reduce the pressure on the intra-prostatic urethra, and thus lead to symptomatic improvement it may take 3–6 months for patients to experience full improvement of their symptoms. Informing patients of this prior to treatment is important, so they do not become discouraged if they do not have immediate relief of symptoms.

## Outcomes and complications

### Urologic outcomes

#### International prostate symptom score

Pisco et al. has published the largest cohort of PAE patients with at least 6 months of follow up (Pisco et al. 2016b). This retrospective study of 630 consecutive patients showed short term (first 12 months), medium term (1–3 years), and long term (3–6.5 years) IPSS improvements by a mean of − 13.71 ± 7.16, − 14.5 ± 7.36, and − 16.94 ± 8.7 respectively (*p* < 0.0001) (Table 3). In a recent meta-analysis of PAE, Kuang et al. 2017, which included 788 patients from the literature, found IPSS improved from a baseline mean of 23.75 to 10.94, 9.31, and 8.90 at 6, 12, and 24 months respectively (*p* < 0.001 for all) (Antunes et al. 2013).

**Table 3:** Prominent papers

	Trial design	Number enrolled	IPSS at 1 year	QoL at 1 year
Pisco et al. (2016b)	Retrospective	630	−13.71	−1.94
UK-ROPE (Ray et al. 2018)	Prospective registry	305 (216 PAE, 89 TURP)	−10.9	−2.6
Gao et al. (2014)	Randomized controlled Trial	114 (57 PAE, 57 TURP)	−13.4	−2.9
Abt et al. (2018)	Randomized controlled Trial	99 (48 PAE, 51 TURP)	−9.23 at 12 weeks	−2.33 at 12 weeks

*IPSS* International prostate symptom score, *QoL* Quality of life, *PAE* Prostate artery embolization, *TURP* Transurethral resection of the prostate

The UK-ROPE study was published this year and evaluated the improvement in IPSS for PAE compared to baseline, as well as to TURP in a non-randomized registry format (Ray et al. 2018). This study enrolled 216 patients in the PAE and 89 in the TURP arms. At the primary endpoint of 12 months PAE demonstrated a significant decrease in IPSS of 10.9, however, less than that of TURP which was 15.2.

Very few randomized controlled trials comparing PAE to other treatments exist. In the largest, Gao et al. randomized 114 patients to TURP (*n* = 57) or PAE (n = 57) (Gao et al. 2014). The authors found that the TURP cohort showed significantly better improvement in IPSS at 1 and 3 months, however, the treatments became equivalent at 6 months and remained equivalent at the 12 and 24 month time points. The initial differences, at 1 and 3 months, may be the result of the need for prostatic tissue to undergo necrosis and tissue remodeling after PAE, for symptoms to abate. In 2018 Abt et al. published another randomized controlled trial comparing PAE and TURP (Abt et al. 2018). This open-label, single center, randomized controlled trial was completed with the primary outcome of non-inferiority of PAE to TURP in terms of IPSS at 3 months. The trial randomized 103 patients, however, they only compared the 48 that underwent PAE to the 51 that underwent TURP. While there was no statistically significant difference in the mean IPSS improvement between the PAE (− 9.23 points) and TURP (− 10.77 points) cohorts at 3 months (*p* = 0.31), non-inferiority of PAE could not be shown secondary to the variation among outcomes (*p* = 0.17).

#### Quality of life

Pisco et al. also demonstrated encouraging improvements in quality of life (QoL) with a mean improvement of − 1.94 ± 1.20, − 1.98 ± 1.21, − 1.74 ± 1.45 at short term, medium term, and long term follow up respectively (*p* < 0.0001 as compared to baseline) (Pisco et al. 2016b). In a recent meta-analysis, the mean QoL had improved from a baseline mean of 4.63 to 2.48, 2.11, and 2.36 at 6, 12, and 24 months respectively (*p* < 0.001 for all) (Antunes et al. 2013).

The UK-Rope trial showed QoL measurements had improved by a mean of 2.6 in the PAE cohort, a marked improvement but not as significant as the TURP cohort’s improvement of 3.4. The study completed a propensity matched analysis for non-inferiority of PAE to TURP which failed to show that PAE was non-inferior in terms of QoL at 12 months (Ray et al. 2018). Abt et al. demonstrated no statistically significant difference in the change in QoL between the PAE (− 2.33) and TURP (− 2.69) cohorts at 3 months (*p* = 0.15) in there randomized controlled trial (Abt et al. 2018). While Gao et al. found that the TURP cohort showed significantly better improvement in QoL at 1 and 3 months, however, the treatments became equivalent at 6 months and remained equivalent at 12 and 24 months (Gao et al. 2014).

#### Maximal flow rate

Pisco et al. demonstrated Qmax also increased significantly over baseline with short term, medium term, and long term follow up patients experiencing a mean improvement of 3.07 mL/s ± 5.84, 4.12 mL/s ± 11.32, and 7.98 mL/s ± 4.83, respectively (*p* < 0.0001). A recent meta-analysis demonstrated Qmax improved from a baseline mean of 8.34 mL/s to 14.26 mL/s, 15.91 mL/s, and 16.91 mL/s at 6, 12, and 24 months respectively.

Abt et al. in there randomized controlled trial did show a statistically significantly better increase in patients Qmax following TURP as compared to PAE (*p* < 0.05), despite PAE patients showing significant improvement over base line (Abt et al. 2018). However, Gao et al. in there randomized controlled trial failed to show statistically significant differences in Qmax improvement after 3 months, with the improvements at 24 months being nearly identical (Gao et al. 2014).

#### Prostate volume and post void residual

Pisco et al. showed PV and PVR improved significantly over base line (*p* < 0.0001) (Pisco et al. 2016b). A recent meta-analysis found significant improvements in PV and PVR over baseline as well (Antunes et al. 2013).

Abt et al. in there randomized controlled trial found that patients PVR was significantly improved from baseline in the PAE cohort, but was statistically inferior to the improvement in the TURP cohort (Abt et al. 2018). However, Gao et al. in there randomized controlled trial failed to show statistically significant differences in PVR improvement after 3 months, with the improvements at 24 months being nearly identical (Gao et al. 2014).

These larger studies highlight the findings that many other authors have documented. The improvement in QoL, Qmax, and IPSS over baseline are significant and while data over 2 years is sparse the treatment has remained durable thus far.

### Complications

Complication profiles are paramount for any new procedure in the BPH induced LUTS space, as TURP is an excellent surgical option in terms of clinical outcomes, however it’s complication profile is what leaves room for improvement. To date the complication profile of PAE has been very promising. Pisco et al. reported 2 major complications (bladder ischemia requiring surgery and persistent perineal pain) in their retrospective review of 630 patients resulting in a major complication rate of 0.3% (Pisco et al. 2016b). Two meta-analyses which included 788 and 840 patients respectively reported major complication rates of 0.4% (3/788) and 0.1% (1/840) respectively (Antunes et al. 2013; Wang et al. 2015). In a randomized controlled trial comparing TURP to PAE, Gao et al. reported that 14.8% (8/54) of PAE and 7.5% (4/53) of TURP patients had major complications (Gao et al. 2014). These results were inconsistent with previously published data, in the number of complications experienced in the PAE group. Gao et al., choose to label technical and clinical failures as major complications, which has been criticized by other authors (Wang et al. 2016). The authors also choose to not consider hemorrhage requiring blood transfusion as a complication in the TURP cohort. If we adjust the Gao data to be more in line with published standards, we find that PAE cohort consisted of no major complications and the TURP cohort experienced major complications in 7.5% (4/53) of patients (Amouyal et al. 2016a). The lack of any retrograde ejaculation, impotence, or incontinence in the TURP group has also raised questions regarding this study’s adverse event reporting and would perhaps further the adverse event margin (Wang et al. 2016). The concerns with the adverse event reporting in Gao has been further supported by the superior complication profile and length of stay demonstrated by a recent randomized controlled trial (Abt et al. 2018) and propensity score matched registry (Ray et al. 2018). Abt et al., in their randomized control trial, found that treatment related adverse events were half as frequent after PAE than TURP (*p* = 0.003). The length of stay was also found to be significantly less in both of these articles (Ray et al. 2018; Abt et al. 2018).

The most common minor complication of PAE has varied depending on the publication. A recent meta-analysis of 840 patients and Pisco et all in there review of 630 patients found that dysuria was the most common minor complication occurring in 10.4% (87/840) and 24.1% (152/630) of patients respectively (Pisco et al. 2016b; Grosso et al. 2015). Kuang et al. 2017 in their meta-analyses of 788 patients found that acute urinary retention was the most common minor complication occurring in 7.61% of cases (60/788) (Antunes et al. 2013). Other commonly seen minor complications included hematospermia, minimal rectal bleeding, and urinary tract infections.

## Future perspectives

As PAE moves from the research to the everyday clinical setting it will be important for the interventionalist community to provide more high level data. First, it should be noted that placebo effect can significantly affect IPSS scores and for this reason most new BPH treatments undergo a placebo trial. This trial would be of great benefit in convincing skeptical, but potentially referring, urologists. Second, when endpoints for future trials comparing PAE to other procedural treatments for medically refractory BPH need to be carefully considered. For instance, when comparing to TURP the primary IPSS improvement endpoint should likely not be before 6 months, given the findings of Gao et al. Also, further attention to perioperative quality of life scores such as the “BPH-6” should be incorporated into future trials. Perioperative scores which incorporate quality of life measures highlight the negative aspects of the current gold standard, TURP, and would shed light on how significantly the improvement could be with PAE.

## Conclusion

Prostate artery embolization is an exciting technique that continues to demonstrate promising results with improving levels of evidence. It is important for interventionalist who wish to start offering this treatment tool to familiarize themselves with basic urologic measurements such as IPSS, urodynamics, and QoL measures as well as learning the technical aspects of the PAE procedure. As with all procedures, patient selection is key to delivering desired results for patients.

## Abbreviations

BPH: Benign prostatic hyperplasia
CTA: Computed tomography angiogram
IPSS: International prostate symptom score
IPSS-S: IPSS storage subscore
IPSS-V: IPSS voiding subscore
LUTS: Lower urinary tract symptoms
MRA: Magnetic resonance angiogram
NSAID: Nonsteroidal anti-inflammatory drugs
PAE: Prostate artery embolization
PSA: Prostate specific antigen
PV: Prostate volume
PVR: Post void residual
Qmax: Maximal flow rate
QoL: Quality of Life
TURP: Transurethral resection of the prostate
